# Three-Dimensional Combustion Field Temperature Measurement Based on Planar Array Sensors

**DOI:** 10.3390/mi17010135

**Published:** 2026-01-22

**Authors:** Xiaodong Huang, Zhiling Li, Jia Wang, Wei Zhang, Yang Liu, Xiaoyong Zhang, Yanan Bao

**Affiliations:** 1 Department of Intelligent and Information Engineering, Taiyuan University, Taiyuan 030051, China; 2024502002@tyu.edu.cn (Z.L.); zbdxzhangwei@163.com (W.Z.); 2023502003@tyu.edu.cn (Y.L.); 2011050004@tyu.edu.cn (X.Z.); 2 School of Semiconductor and Physics, North University of China, Taiyuan 030051, China; wangjianuc2022@163.com; 3 Shanxi Key Laboratory of Advanced Semiconductor Optoelectronic Devices and System Integration, Jincheng 048000, China; baoyanan@jcgjd.org.cn; 4 Jincheng Research Institute of Opto-Mechatronics Industry, Jincheng 048000, China

**Keywords:** temperature measurement, TDLAS, area array sensor, three-dimensional imaging

## Abstract

High-resolution three-dimensional temperature fields are essential for studying flame combustion, and tunable diode laser absorption tomography (TDLAT) is an effective method for diagnosing flame combustion conditions. In actual combustion measurements, the reliance of TDLAT on line-of-sight (LOS) measurements leads to limited data and reduced dimensionality in analyzing combustion fields. This study proposes a method using area-array sensor-coupled absorption spectroscopy to measure the three-dimensional temperature field of flame accurately, aiming for enhanced combustion diagnosis. The laser beam is configured into a cone shape, and after traversing the combustion field under examination, the area-array sensor receives a projection signal. This signal is then used to reconstruct a high-resolution, multidimensional temperature field. We confirmed the accuracy and robustness of the algorithm through numerical simulations and compared these with experimental results from the TDLAT setup. Our TDLAT detection system demonstrates high precision and effectively measures temperature fields in complex flame imaging scenarios.

## 1. Introduction

Turbulent disturbances during combustion significantly affect the accuracy of flame measurements, presenting a challenge for current diagnostic technologies. While these technologies strive to enhance measurement precision, they often fall short in accurately addressing the complexities introduced by turbulence. Temperature, being a critical parameter in understanding combustion processes [1,2,3], highlights the necessity for more refined measurements. High spatial and temporal resolution is essential for this refinement. Improving resolution directly boosts the system’s imaging capabilities, allowing for the capture of detailed object information. This enhancement enables researchers to analyze combustion conditions with greater accuracy, leading to more precise diagnostics in combustion studies.

Intrusive temperature measurement techniques, such as thermocouples and gas sampling tools, are practical for measuring temperature and gas molar concentration [4,5]. However, these refined measurement methods for flames can inadvertently alter combustion characteristics and potentially distort the measurement outcomes. Noncontact optical temperature measurement methods utilize laser and radiation spectroscopy, including interference spectroscopy [6,7], coherent anti-Stokes Raman scattering spectroscopy (CARS) [8,9], absorption spectroscopy [10,11], monochromatic [12,13], and multispectral [14,15,16]. These methods are highly accurate and noninvasive and can quantitatively analyze the combustion flame and evaluate its true combustion state. Consequently, they are extensively used in various domains such as thermal power plants, aerospace engine development, military applications, and several other fields [17,18,19]. Among these techniques, tunable diode laser absorption tomography (TDLAT) stands out with its noncontact nature, high sensitivity, and rapid response time [20,21,22]. This makes it a crucial tool for the combustion diagnosis in internal combustion engines and ramjet engines [23,24].

Wen and Wang adopted a multiline temperature-measurement approach, using TDLAS technology to determine the spatially- and temporally resolved temperatures in counterflow diffusion flames [25]. Through the measurement of four counterflow flames and employing the hedge diffuse flame module in Chemkin for numerical prediction, they confirmed that the observed temperature curves were consistent with the predicted temperature curves across all test flames. Wang and Deguchi utilized 32-path TDLAT technology to reconstruct the two-dimensional (2D) temperature and concentration profiles within a pulverized coal furnace [20]. This was achieved under challenging conditions of high temperature, dust, humidity, and corrosion, and the results were integrated with a CFD-simulated flame. Subsequently, the temperature and water vapor (H_2_O) concentration were compared to validate the reliability of the system for 2D temperature measurement. Xu et al. and others developed a TDLAS detection system incorporating a five-viewpoint fan-shaped laser beam-sensing array [26,27,28,29]. They employed techniques such as TDLAT and multispectral line temperature measurement to reconstruct 2D temperature and H_2_O concentration profiles of the combustion flame. Accordingly, a single spectral line method was proposed to extract the gas temperature and concentration profiles. The distributions of the flame temperature and internal H_2_O concentration were reconstructed using the line shape of a single absorption spectrum to simultaneously decouple the gas parameters along the laser path. Spearrin et al. designed a mid-infrared (mid-IR) laser absorption imaging (LAI) system for reconstructing gas temperature and carbon monoxide (CO) concentration in laminar flames [30]. This experimental system was noted for its complex configuration. In practical engineering scenarios such as aero-engines, gas turbines, and thermal power plants, the complexity of the testing environment makes it challenging to deploy adequate detection equipment and obtain effective data for reconstructing the original distribution. This limitation is a significant impediment to the advancement of TDLAS.

In this study, we propose a method using area-array sensor-based TDLAT to measure the spectral information of oxygen (O_2_) in combustion reactions and reconstruct the flame temperature distribution. First, by conducting a temperature calibration experiment with the detection system, we derived a mathematical relationship between the integrated absorbance of O_2_ molecules and temperature. This relationship was then applied to measure the flame temperature using the detection system. Through numerical simulation, the reliability of the algorithm was verified, and the reconstruction error was found to be less than 7%. The three-dimensional (3D) temperature distribution of the flame was then reconstructed by combining the calibration results and filtered back-projection algorithm. The accuracy of the reconstruction was confirmed through temperature measurements using thermocouples, yielding a maximum relative error of 5.16%. The approach presented in this work demonstrates a method for developing high-precision flame temperature imaging technology.

## 2. Models and Methods

### 2.1. Absorption Spectrum Model

In the TDLAT temperature measurement setup [31], the laser was modulated by a signal generator to produce a narrowband incident light intensity *I*_0_. The laser wavelength covers the absorption peak of the gas molecules to be measured. As the incident light traverses the area under measurement, it resonates with the target molecules undergoing a transition. This process leads to the absorption of the incident light energy, resulting in attenuated outgoing light intensity It, which is then detected by an optical sensor. According to the Beer–Lambert law [32], the functional expression of the transmission coefficient *τ*(*ν*) of the laser center frequency ν (cm^−1^) can be expressed as

(1) $$\tau (\nu )=\frac{I_{t}}{I_{0}}=\exp\left\{-\int_{0}^{L}P(x)\cdot X(x)\cdot S[T(x)]\cdot \phi (\nu )dx\right\}$$

where *L*(cm) is the absorption optical path length, *P*(atm) is the environmental pressure of the area under measurement, *X* is the molar concentration of the absorbing component, *S*(*T*) is the intensity of the spectral line, *T*(*K*) is the temperature of the gas, and the line shape function *ϕ*(ν) was determined by temperature, pressure, and gas concentration.

If the flow field under measurement is uniform, Equation (1) can be simplified as

(2) A=PXL·S(T)

where A is the absorption-integrated absorbance (cm^−1^). Among TDLAT technologies, direct absorption spectroscopy (DAS) is a widely used technique, featuring simple operation and convenient processing characteristics. Accordingly, two different absorption spectral lines of the same gas molecule can be used to calculate parameters such as the flow field temperature, component content, and pressure. This is called dual-line measurement method, as follows:

(3) $$R(T)=\frac{A_{1}}{A_{2}}=\frac{S_{1}(T)}{S_{2}(T)}=\frac{S_{1}(T_{0})}{S_{2}(T_{0})}\exp[-\frac{hc}{k}({E_{1}}^{\prime \prime }-{E_{2}}^{\prime \prime })(\frac{1}{T}-\frac{1}{T_{0}})]$$

In Equation (3), Si(*T*_0_) is the gas absorption spectrum line intensity at the reference temperature *T*_0_. In the HITRAN database, *T*_0_ = 296 K, *h* is the Planck’s constant, *c* is the speed of light, *E*′′ is the low-level transition energy of the gas, and *k* is Boltzmann constant. The integrated absorbance ratio of the two absorption spectral lines is a single-valued function of temperature. Hence, the temperature of the combustion field under measurement can be calculated using the measured integrated absorbance, as follows:

(4) $$T=\frac{\frac{hc}{k}({E_{1}}^{\prime \prime }-{E_{2}}^{\prime \prime })}{\ln(\frac{A_{1}}{A_{2}})+\ln(\frac{S_{2}(T_{0})}{S_{1}(T_{0})})+(\frac{hc}{k})(\frac{{E_{1}}^{\prime \prime }-{E_{2}}^{\prime \prime }}{T_{0}})}$$

### 2.2. Algorithm Model

The TDLAT can provide high-quality, high-resolution projection data. Among reconstruction algorithms, the computational efficiency of the filtered back projection (FBP) algorithm is higher than that of iterative algorithms such as algebraic, multiplicative, and simultaneous algebraic reconstruction techniques. Although the FBP algorithm is sensitive to noise and missing data, it typically provides sufficient projection data to mitigate these issues. After comprehensive considerations, we chose the FBP algorithm to process the subsequent data.

**Figure 1:**
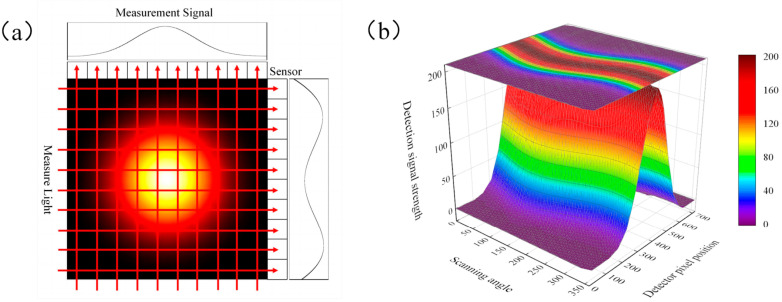
Schematic of the TDLAT measurement projection. (**a**) Measurement signal diagram. (**b**) Scanning waveform distribution intensity diagram.

The basis of the TDLAT reconstruction is to obtain the projection signal, as shown in Figure 1a, the 2D distribution information was projected onto a one-dimensional (1D) linear array, similarly, the 3D distribution information was also projected onto a 2D plane. The mathematical model employed is expressed by Equation (5), where *I* is the laser intensity, *α* is the photoelectric conversion coefficient, and *G* is the gray value of the area array sensor. The projection data were obtained through calculations, and the distributions of various physical quantities in the area under measurement were inverted. For each plane, the measurement signal obtained by the linear array detector was the projection of the plane distribution information in the vertical angular direction of the linear array detector. Taking the line-detector measurement projection waveform of the Gaussian function image area in Figure 1a as an example, the scanning angle varied from 0° to 359°, the step angle was 1°, and a set of detection signals was obtained at each angle. The intensity distribution is depicted in Figure 1b, and the 2D image of the scanning result is presented as a projected sinogram.

(5) $$\left[\begin{array}{cccc} I_{11} & I_{12} & \ldots & I_{1n}\\ I_{21} & I_{22} & \ldots & I_{2n}\\ \vdots & \vdots & \ddots & \vdots \\ I_{n1} & I_{n2} & \ldots & I_{nn} \end{array}\right]\left[\begin{array}{c} \alpha_{1}\\ \alpha_{2}\\ \vdots \\ \alpha_{n} \end{array}\right]=\left[\begin{array}{c} G_{1}\\ G_{2}\\ \vdots \\ G_{n} \end{array}\right]$$

The quality of the scanning sinogram obtained is influenced by factors such as the scanning angle, step angle, and the number of detector pixels. These factors directly impact the spatial and angular resolutions of the sinogram. Utilizing the acquired sinogram image, an algorithm was employed to reconstruct the area information. The theoretical foundation of FBP is based on Fourier’s slicing theorem [33,34]. The FBP method features advantages such as high accuracy, high speed, and good real-time performance. The projection signal *F*(ω) is filtered by the filter of the transfer function *H*(ω) to obtain the modified projection *G*(ω), as expressed in Equation (6), which is a fixed-point ray equation.

(6) G(ω)=F(ω)∗H(ω)

In FBP, all filtered projections of a fixed point are accumulated over a range of 0–π for back-projection reconstruction, resulting in the determination of the pixel value. Therefore, in this study, the reconstruction process of this algorithm was divided into the following three steps [35]:

(1) Filter the projection *p*(*x*,*φ*) measured at a fixed viewing angle *φ* to obtain the filtered projection *g*(*x*,*φ*);
(2) Back-project *g*(*x*,*φ*) under each *φ* across all points on the ray satisfying *x* = −cos(θ − φ);
(3) Accumulate the back-projection values in step 2 for all 0 < *φ* ≤ π, and finally obtain the reconstructed image.

The critical part of FBP is the window filter function. In this study, we utilized an enhanced version of the Shepp–Logan (S-L) window filter function [36]. The focus of the S-L window filter function is the slow cutoff in the frequency domain. Its function is expressed as

(7) $$g(\omega )=\frac{\omega }{\pi }\times \frac{\sin(\omega /2)}{\omega /2}$$

where *ω* represents the frequency of the filter window function, and *g*(*ω*) represents the function value of the filter window function. The S-L window filter function is relatively smooth and can suppress high-frequency components in the projection, thereby reducing the oscillatory response in the image reconstruction. Upon comparison, it was observed that the reconstruction quality was better for projection data containing noise. A drawback of this filter window function is its limited image reconstruction quality at low frequencies, which can potentially impact the recovery and overall quality of the image to some degree. Consequently, the S-L filtering window function was enhanced by incorporating weighted smoothing method, which improved the smoothness of the central area. This method effectively enhances the retention of low-frequency information by assigning higher smoothing weights to the central region, thereby significantly improving the image reconstruction quality. This step focuses on optimizing the processing of the central area of the image, ensuring better preservation of low-frequency components. This is crucial for enhancing the overall quality of the image reconstruction.

### 2.3. TDLAT Measurement System and Calibration Experiment

**Figure 2:**
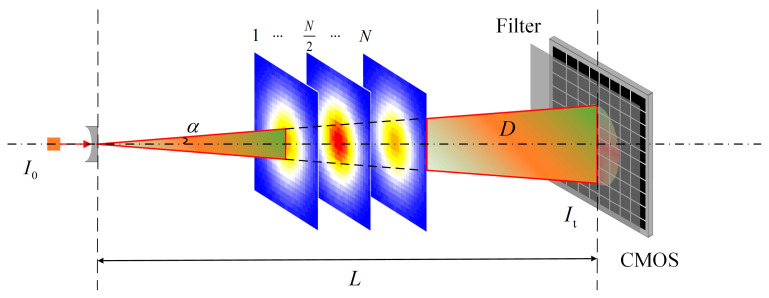
Schematic of the experimental optical path.

Silicone photodetectors are used in classic TDLAT measurement systems. However, in most practical experiments, the issue of point distribution remains unresolved, necessitating the collection of additional data for accurate temperature field reconstruction. Therefore, this study employed a high-resolution area-array sensor (ISDI-3131, ISDI, London, UK) featuring a spatial resolution of 100 μm, a frame rate of 100 frames per second (FPS), and a 500 × 500 pixel area for data sampling. The experimental optical path is illustrated in Figure 2. After the temperature field was discretized and sliced, a cone-shaped laser beam was passed through. The area-array sensor detects the field being measured, enabling the acquisition of extensive photoelectric information with limited equipment.

**Figure 3:**
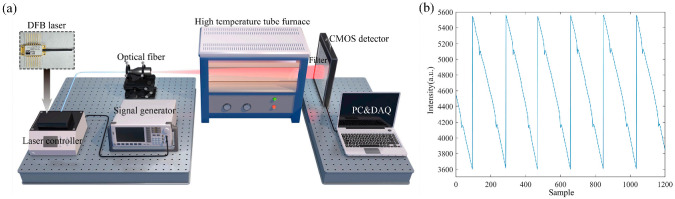
TDLAT calibration experiment. (**a**) Calibration system diagram. (**b**) Oxygen (O_2_) molecular absorption curve graph.

The TDLAT calibration system is shown in Figure 3a. The system was composed of a signal generator, laser driver, DFB laser, and cone beam shaping module. The temperature range of the high-temperature tube furnace was set at 25 °C to 1500 °C. Upon configuring the necessary optical parameters and calibration distance, the temperature control device was used to adjust the temperature range between 300 °C and 1000 °C. Based on the prior laser calibration experiment and referencing the HITRAN database [37], wavelengths of 760.77 nm and 760.88 nm were chosen as the absorption spectral lines. The laser emitted light with intensity *I*, which, after passing through the optical-shaping module, formed a cone-shaped beam. This beam then traversed the constant-temperature area and was subsequently detected by the area-array sensor. The initial data depicting O_2_ molecule absorption at 573.15 K is presented in Figure 3b.

**Figure 4:**
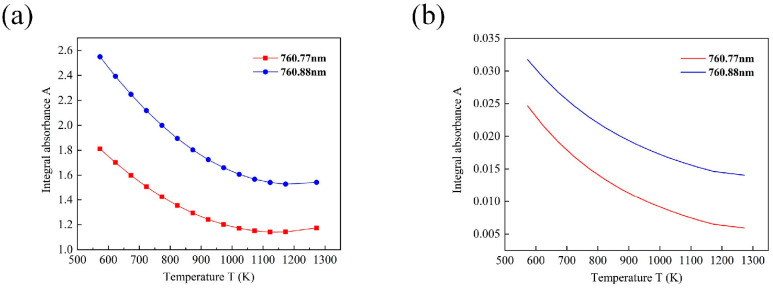
Integrated absorbance plot of oxygen (O_2_) molecules. (**a**) Plot of integrated absorbance *A*. (**b**) Plot of HITRAN database integrated absorbance simulation.

The sawtooth wave absorption signal underwent multiple processing steps, during which a nonabsorption area was selected to establish the nonabsorption baseline. Following the Gaussian line fitting of the measured spectral signal, the integrated absorbance was calculated. The same method was used to process the O_2_ molecules at different temperatures. The original absorption signal was acquired, and from this, the fitting curve correlating temperature *T* with integrated absorbance *A* was derived, as illustrated in Figure 4a. Their relationships are expressed as follows:

(8) $$A_{1}=\left(2.051\times 10^{-6}\right)T^{2}-\left(3.58\times 10^{-3}\right)T+2.703$$

(9) $$A_{2}=\left(2.623\times 10^{-6}\right)T^{2}-\left(4.85\times 10^{-3}\right)T+3.769$$

where *A*_1_ is the integrated absorbance of O_2_ molecules at 760.77 nm, *A*_2_ is the integrated absorbance at 760.88 nm, and *T* is the temperature. The integrated absorbance of two wavelengths simulated from the HITRAN database is shown in Figure 4b. Compared with the calibration experiment, it is found that the curve change of the calibration experiment is the same as that of the database, so the fitting is effective.

## 3. Numerical Simulations and Experiments

To evaluate effectiveness of the modified FBP algorithm in reconstructing 3D temperature fields, we carried out numerical simulations along with butane (C_4_H_10_) flame temperature field reconstruction experiments. The numerical simulations were used to validate the feasibility, noise resistance, and overall performance of the algorithm in reconstructing temperature fields. We also assessed the effects of varying complexities on temperature field reconstruction, as well as the impact of noise and different algorithms. Three indicators were selected as the algorithm model assessment criteria: Peak signal-to-noise ratio (PSNR), structural similarity (SSIM), and temperature reconstruction relative error (Err). These definitions are given as

(10) $$MSE=\frac{1}{mn}\sum_{i=0}^{m-1}\sum_{j=0}^{n-1}{\left[I(i,j)-K(i,j)\right]}^{2}$$

(11) $$PSNR=10\cdot \log_{10}(\frac{MAX_{I}^{2}}{MSE})=20\cdot \log_{10}(\frac{255}{\sqrt{MSE}})$$

where m and n are the length and width dimensions of the image, MSE is the mean square error, and MAX*_I_* is the pixel range of the image (1 or 255). The SSIM is presented as

(12) $$SSIM(x,y)=\frac{\left(2\mu_{x}\mu_{y}+c_{1})\cdot (2\sigma_{xy}+c_{2}\right)}{\left(\mu_{x}^{2}+\mu_{y}^{2}+c_{1})\cdot (\sigma_{x}^{2}+\sigma_{y}^{2}+c_{2}\right)}$$

where *x* and *y* are the original and reconstructed images, $u_{x}$ and $u_{y}$ are the average values corresponding to the images *x* and *y*, $\sigma_{x}^{2}$ and $\sigma_{y}^{2}$ are the variances corresponding to images *x* and *y*, $\sigma_{xy}$ is the covariance of *x* and *y*, $c_{1}={\left(k_{1}L\right)}^{2}$ and $c_{2}={\left(k_{2}L\right)}^{2}$ are constants used to maintain stability.

The reconstruction error compares the reconstructed temperature distribution result, *T^cal^*, with the actual value, *T^real^*. This comparison is used to calculate the relative error of the reconstructed temperature field, denoted as err.

(13) $$err=\frac{\left|T^{cal}-T^{real}\right|}{T^{real}}\times 100\%$$

### 3.1. Numerical Simulations

Numerical simulations were conducted to verify the feasibility of the proposed algorithm and its ability to reconstruct complex data. The distribution model is constructed as a 3D Gaussian function, as follows:

(14) $$f\left(x,y,z\right)=A\exp\left(-\left(\frac{{\left(x-x_{0}\right)}^{2}}{2\sigma_{x}^{2}}+\frac{{\left(y-y_{0}\right)}^{2}}{2\sigma_{y}^{2}}+\frac{{\left(z-z_{0}\right)}^{2}}{2\sigma_{z}^{2}}\right)\right)$$

where $\left(x_{0},y_{0},z_{0}\right)$ is the mean (or center coordinate) on the *x*, *y* and *z* axes, *σ_x_*, *σ_y_*, and *σ_z_* are the standard deviations on the *x*, *y* and *z* axes. The “width” of the Gaussian distribution is correlated with its value; a larger value indicates a wider distribution.

**Figure 5:**
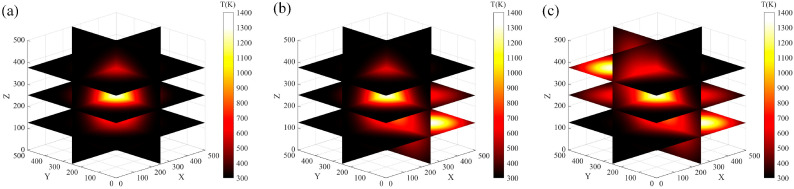
Original temperature field. (**a**) Single Gaussian function distributions. (**b**) Double Gaussian function distributions. (**c**) Triple Gaussian function distributions.

We used Gaussian function distributions 1, 2, and 3 to verify the accuracy of the proposed algorithm. The space size was set to 500 × 500 × 500 pixels using 18 angles at 20° intervals. A 3D reconstruction of the three Gaussian distributions was performed, as shown in Figure 5.

**Figure 6:**
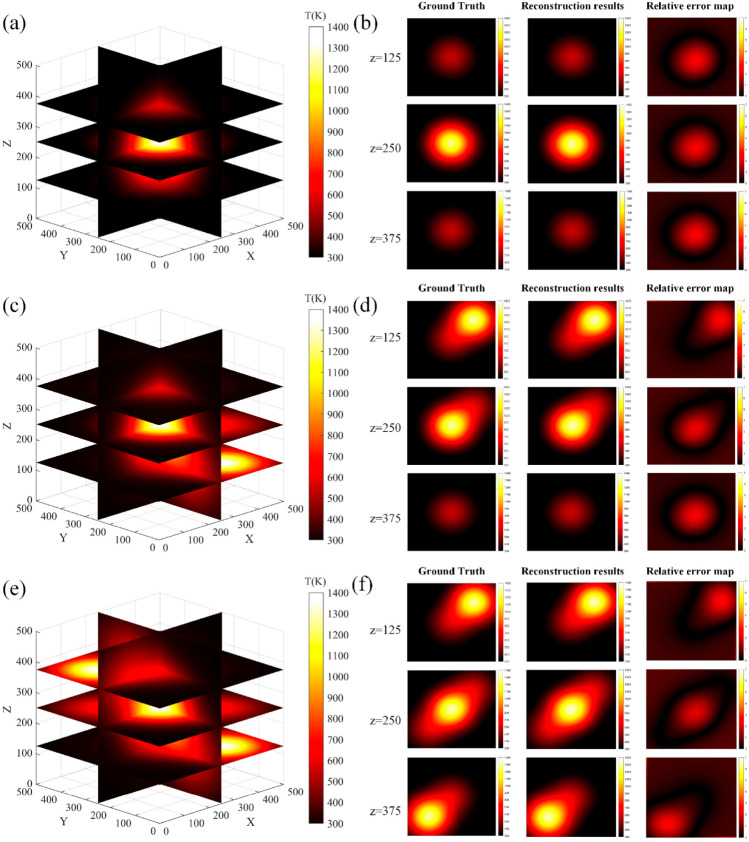
Temperature field reconstruction slices. (**a**,**b**) depict 3D slices of a single temperature field, along with 2D slices and relative error distributions at heights *z* = 125, 250, and 375. (**c**,**d**) show 3D slices of dual temperature fields, including 2D slices and relative error distributions at the same heights. (**e**,**f**) present 3D slices of three distinct temperature fields, accompanied by 2D slices and relative error distributions at heights *z* = 125, 250, and 375.

The reconstructed 3D slices for the three temperature fields are displayed in Figure 6a,c,e. At three different heights, specifically *z* = 125, 250, and 375, original temperature field slices were taken for 2D reconstruction, and their relative errors were calculated. The corresponding results are presented in Figure 6b,d,f.

From the 3D slices, it is evident that the improved FBP algorithm effectively reconstructs three types of Gaussian function distributions, clearly distinguishing between the Gaussian function distribution and non- Gaussian function distribution areas. The 2D slice diagrams reveal that errors are primarily concentrated at the boundaries, while the regions inside and outside the Gaussian function distribution exhibit minimal errors. The maximum relative errors observed were 2.76% for the single Gaussian function distributions, 4.11% for the double Gaussian function distributions, and 6.16% for three Gaussian function distributions, indicating an increase in error with rising the number of Gaussian functions. In this study, both PSNR and SSIM were calculated, with PSNR exceeding 45 dB and SSIM greater than 0.99, demonstrating a favorable reconstruction effect.

### 3.2. Anti-Noise Experiments

**Figure 7:**
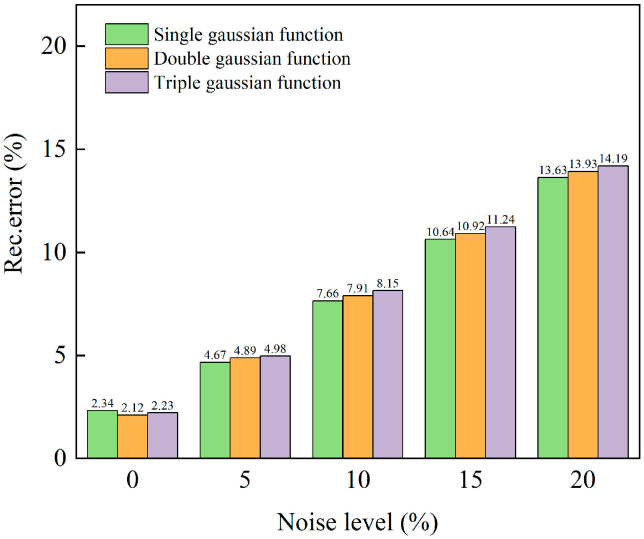
Reconstruction relative error diagram of three Gaussian function distributions under different noise levels.

In practical applications, real combustion fields are very complex. While incorporating a specific proportion of noise into the numerical simulation’s original Gaussian function distributions does not fully replicate the signal noise in actual measurements, the added noise is random. Due to its randomness and uncertainty, it partially mimics the noise encountered in real-life scenarios. In this study, a random noise of 0% to 20% was added to the three types of Gaussian function distributions to verify the anti-noise ability of the algorithm. We selected a section with a height of *z* = 250 for Gaussian function distributions reconstruction. The relative errors of the three types of Gaussian function distributions under different random noise levels are shown in Figure 7. It is evident that an increase in the number of Gaussian function has a minimal effect on the error. The error progressively increases as the noise level rises. With a 10% random error, the maximum relative error between the reconstructed and original Gaussian function distributions is 8.15%, indicating that the reconstructed Gaussian function distributions is more consistent with the original Gaussian function distributions. At a random noise level of 20%, the maximum relative error reaches 14.19%, primarily due to individual outliers in the edge area of the Gaussian function distributions. Nevertheless, the overall distribution of the Gaussian function distributions is still accurately represented. The PSNR values exceed 33.79 dB and the SSIM values are above 0.82, indicating that the improved FBP algorithm employed in this study demonstrates strong robustness.

### 3.3. Combustion Flame Experiments

**Figure 8:**
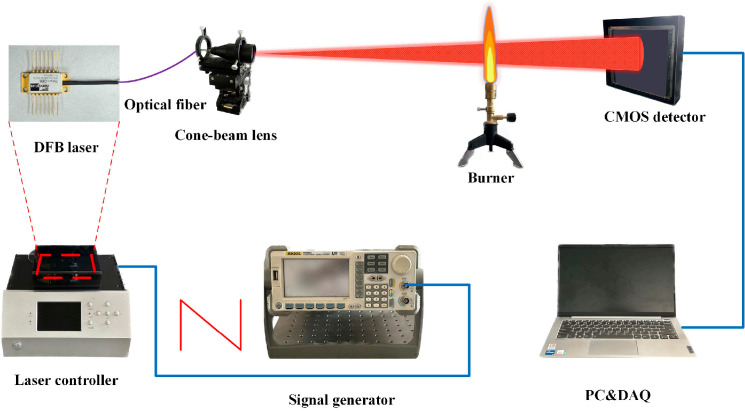
TDLAT measurement system employed in this study.

In this research, a novel TDLAT system and the direct absorption method were utilized to conduct measurement experiments within a combustion flame for reconstructing the 3D temperature field. The measurement setup, as depicted in Figure 8, includes a signal generator, laser driver, DFB laser, collimating mirror, cone-beam shaping module, and an area-array sensor. The O_2_ molecules in the flame area of the Bunsen burner were measured. The Bunsen burner, utilizing butane as fuel and air as the oxidant, operated at a gas flow rate of 0.76 L/min. The sample was fully burned after premixing. The signal generator produced a sawtooth wave, which was then fed into a laser driver. This driver controlled the laser to emit light at two specific wavelengths (760.77 nm and 760.88 nm). The laser light, after passing through a collimator and a beam-shaping module, formed into a cone beam. This beam then interacted with O_2_ molecules in the flame area of the burner. The rotary displacement stage of the burner rotated the flame by 20° for each measurement. An area-array sensor collected spectral signals corresponding to changes in laser light intensity at 18 different angles, the collection time was 36 s. After multiple collections, these signals were processed on a computer, which then reconstructed the temperature distribution of the flame.

**Figure 9:**
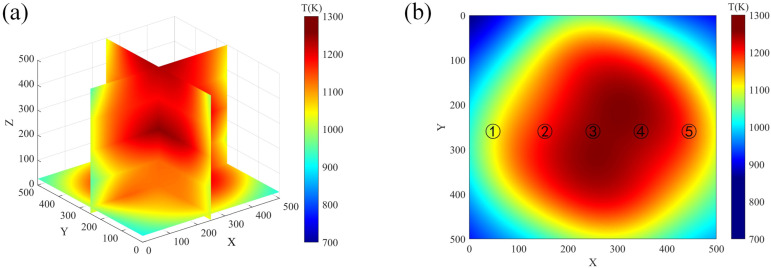
Three-dimensional (3D) temperature field of flame. (**a**) Sectional view of 3D temperature field. (**b**) Sectional view of *z* = 250.

After processing the data, a 2D temperature projection of the combustion flame at 18 different angles was acquired, and an enhanced FBP algorithm was applied for its reconstruction. Figure 9a displays a cross-section of the reconstructed 3D temperature field, with the peak temperature reaching 1259 K. It is observed that the maximum temperature of the combustion flame initially increases and then decreases with height, with the center temperature being closest to the *z* = 250 section. Various methods for verifying flame temperature include simulated flames, infrared thermal imaging cameras, and thermocouples. In this study, modified K-type thermocouples were utilized at the plane of height *z* = 250, with 5 measurement points as illustrated in Figure 9b. These sampling points were measured and compared with the reconstructed 3D temperature field of the flame to validate the accuracy of the reconstruction.

The relative errors of the measurements are listed in Table 1. The maximum error between the reconstructed results and actual measurements is 5.16%. This deviation could stem from integration errors during the temperature field calculation, though it is important to note that these findings are still in the verification stage. The lower thermocouple measurements, compared to the reconstructed temperature field, can be attributed to factors such as thermal inertia, heat conduction loss, and system errors, which diminish the flame resistance and impact measurement accuracy. Nonetheless, temperature imaging techniques that utilize area-array sensors in conjunction with TDLAT demonstrate high accuracy and reduced system complexity.

## 4. Conclusions

This study proposed and demonstrated a method for 3D flame temperature imaging using a high-resolution area-array sensor in conjunction with TDLAT technology. The novel TDLAT system enhances spatial resolution even with a limited number of detectors, effectively addressing the common issue of insufficient datasets. The refined FBP algorithm delivers higher accuracy and speed in reconstructing multidimensional temperature fields. Numerical simulations revealed that the maximum reconstruction error in the three Gaussian function distributions was 6.16% and the minimum error in the single Gaussian function distributions was 2.76%. Increasing the number of Gaussian functions has little effect on the results; however, higher noise levels correspond to increased reconstruction errors. Under the 20% noise condition, the maximum error recorded was 14.19%, demonstrating the algorithm’s robustness. The ability of the improved FBP algorithm to reconstruct Gaussian function distributions under various noise conditions highlights its strong reconstructive capabilities and effectiveness in terms of accuracy and noise resistance. In practical 3D combustion field-reconstruction experiments, thermocouple measurements verified the numerical results, with a relative error within 6%, underscoring the algorithm’s efficiency in representing actual combustion temperature fields. Therefore, the proposed innovative TDLAT detection system, coupled with the enhanced FBP algorithm, elevates its practical applicability in measurements and offers a method for high-resolution TDLAT measurement imaging.

## Figures and Tables

**Table 1:** Error analysis based on thermocouple.

Position	Thermocouple Measurements (K)	Reconstructed Value (K)	Relative Error (%)
1	1054	1099	4.27
2	1143	1202	5.16
3	1196	1252	4.68
4	1193	1250	4.78
5	1126	1179	4.71

## Data Availability

The data presented in this study are available in this article.
